# Stochastic process-based drought monitoring and assessment system: A temporal switched weights approach for accurate and precise drought determination

**DOI:** 10.1371/journal.pone.0307323

**Published:** 2025-02-06

**Authors:** Muhammad Asif Khan, Sergey Barykin, Dmitry Karpov, Nikita Lukashevich, Akram Ochilov, Rizwan Munir

**Affiliations:** 1 Earth System and Global Change Lab, School of Environmental Science and Engineering, Southern University of Science and Technology, Shenzhen, PR China; 2 Graduate School of Service and Trade, Peter the Great St. Petersburg Polytechnic University, St. Petersburg, Russia; 3 Peter the Great St. Petersburg Polytechnic University, St. Petersburg, Russia; 4 Graduate School of Industrial Management, Peter the Great St. Petersburg Polytechnic University, St. Petersburg, Russia; 5 Karshi State University, Karshi, Uzbekistan; 6 School of Statistics and Data Science, Jiangxi University of Finance and Economics, Nanchang City, Jiangxi Province, PR China; RMIT University, AUSTRALIA

## Abstract

Drought is a recurring climate phenomenon that naturally occurs in all climate regions and leads to prolonged periods of water scarcity. The primary cause of water shortages is inadequate precipitation, which can be influenced by meteorological factors such as temperature, humidity, and precipitation patterns. Effective drought mitigation policies necessitate the monitoring and prediction of drought. To determine the severity and impacts of droughts accurately and precisely, probabilistic models have been developed. However, erroneous drought detection with probabilistic models is always possible. As a result, a novel system for meteorological, agricultural, and hydrological droughts based on the Stochastic Process (Markov chain (MC)) has been proposed to address this issue. The proposed method incorporates the Multi-Scalar Seasonally Amalgamated Regional Standardized Precipitation Evapotranspiration Index (MSARSPEI) for timescales 1–48 and employs temporal switched weights. These weights are generated from the Transition Probability Matrix (TPM) of each temporal classification of the drought type in accordance with the MC’s fundamental assumption. The developed system was implemented on nine meteorological stations in Pakistan. By leveraging historical data and information, the system enables the categorization of droughts. The resultant classifications can be incorporated into effective drought monitoring systems, which can help in devising specific policies to alleviate the effects of droughts.

## Introduction

The occurrence of drought is a natural calamity that causes considerable disruption to human activities, ecosystems, natural systems, and water supply [1, 2]. In recent decades, droughts have become increasingly common, particularly in Pakistan (e.g., *1998–2002*, *2004–2005*, *and 2009*), and they may become worse during the 21^st^ century [3]. Drought and its negative consequences are of utmost concern to policymakers in Pakistan, as agriculture is the backbone of the country’s economy. Defining drought is challenging due to its insidious nature, and various definitions have emerged due to differences in socioeconomic conditions, hydro-meteorological variables, and high water demand [4–6]. Drought is a common occurrence, and its effects accumulate over time. Inadequate precipitation, caused by several meteorological factors, is also thought to cause water shortages [4, 7]. In addition, the probability of recurrent droughts is heightened due to climate change and ongoing global warming. Meteorological drought refers to a situation with a deficiency in rainfall or precipitation for an extended period, ranging from several weeks to years [8, 9]. Agricultural drought is when soil moisture levels are inadequate to support plant or crop growth and maintain grazing pastures. This occurs when the soil moisture content falls below the normal annual average, reducing crop yield. Hydrological drought is a phenomenon that arises due to prolonged precipitation deficits, leading to a reduction in the availability of surface or subsurface water resources. This reduction, in turn, results in groundwater depletion, streamflow, lake, and reservoir levels. Unlike meteorological droughts that may come to an end, hydrological droughts can continue for an extended period of time.

Natural catastrophes usually proclaim their arrival: e.g., Tornadoes roar, hurricanes uproot trees, and wildfires destroy entire landscapes. These huge, uncertain occurrences trigger devastation when they strike and disappear. However, drought is different; it usually does not make any big entrance -the beginning of the drought might yet be inaccurate for a bit of a dry period- and over time its impact builds. Drought, referred to as a “creeping catastrophe,” leaves a devastation trail just as dangerous and deadly as any other severe weather occurrence [10]. Drought has impacted more people worldwide than any natural disaster in the last four decades. Moreover, drought can have significant social, political, economic, and health effects with extensive consequences. Approximately $8 billion is spent annually on resolving the adverse effects of drought hazards [11]. Due to their complex nature and extensive effect, droughts have gained consideration from researchers, and drought analysis has made considerable progress. Various methods have been proposed to assess drought conditions, including drought indices, widely used for monitoring and prediction.

Drought indices are quantitative measures that define the drought event’s duration, frequency, and intensity. In earlier research, numerous drought indices were proposed to assess meteorological, hydrological, and agricultural drought [12–14]. Additionally, Markov Chain (MC) and Transition Probability Matrix (TPM) are powerful tools for drought analysis, providing accurate predictions of drought progression and severity. Recently, these methods have been utilized for drought forecasting [15], risk assessment [16], monitoring and early warning systems [17], and analysis of drought persistence [18]. These studies have shown promising results in drought analysis, providing valuable information for drought management and planning.

This study proposes a new drought analysis and assessment scheme that uses MC models to define meteorological, agricultural, and hydrological drought; the details of the selected stations can be seen in [19]. The model can jointly classify the region under investigation based on various timescales (1–48). The proposed scheme classifies timescales 1–3 for meteorological drought, timescales 1–6 for agricultural drought, and timescales 6–48 for hydrological drought, respectively. Sections 2 and 3 describe the proposed scheme, Section 4 depicts the results and discussion, whereas Section 5 presents the conclusion of the current study.

## Methods

The MC was chosen for the current study due to its robustness in modeling the sequential and stochastic processes, which is critical for understanding and forecasting climatic phenomena such as droughts. MCs function on the presumption of finite memory, unlike several other probabilistic models, for instance, Bayesian networks, which demand previous knowledge and intricate conditional linkages. This implies that the current condition alone determines future states, not the chain of events that led up to them. This characteristic renders MC appropriate for simulating drought scenarios in which the current climate dominates future weather patterns.

Moreover, a detailed outline of the existing methodology and the structure of the proposed drought index can be seen in the subsequent subsections. The study was conducted in accordance with the ethical principles set forth by the responsible committee on human experimentation and adhered to the latest (2008) version of the Helsinki Declaration of 1975.

### Multi-scalar Standardized Drought Indices (SDIs)

The Standardized Precipitation Index (SPI) was first introduced by [20] as a method of assessing precipitation deficiency over different time scales (e.g., 1-, 3-, 6-, 9-, 12-, 24 months) using long-term precipitation records. It is based on the long-term precipitation series and can be calculated by standardizing the appropriate probability density function (pdf) of the cumulative precipitation record using the concept of standard inverse Gaussian functions. The values of SPI can be positive or negative, indicating greater or less than average precipitation, respectively [21]. The expert team at the “Lincoln Declaration on Drought Indices” meeting suggested the use of SPI by National Meteorological and Hydrological Services (NMHs) worldwide for characterizing meteorological drought [22]. Additionally, due to probabilistic and simple mathematical structure—in the past- numerous applications of drought assessment were SPI-based. The standardized Precipitation Evapotranspiration Index (SPEI) was first proposed by [23] as a drought index that takes into account both precipitation and temperature records. The calculation of SPEI uses the water balance model (deficient D) similar to SPI, where D is the series of differences between precipitation and Potential Evapotranspiration (PET). However, [23] used the Thornthwaite equation to calculate PET, which can lead to under or over-estimation of PET in areas with low temperatures [24]. Several other methods are available for accurate PET series estimation, such as the Penman equation and the Blaney-Criddle method [25]. Various studies have used SPEI for drought assessment, including individual applications of SPEI, rather than comparing it with SPI [26–28]. In recent studies, SPTI (Standardized Precipitation Temperature Index) has also been proposed as a new drought index [29]. Unlike SPEI, SPTI uses the Demartone aridity index to quantify its values and shows a significant correlation with SPI in areas with low temperatures, making it a good candidate for multivariate drought assessment. However, in this study, the Hargreaves (HS) equation was used to estimate PET, utilizing the “Hargreaves function” of the SPEI R package. The Hargreaves equation is suitable for areas with low temperatures, unlike Thornthwaite’s equation.

### Drought index using a Markov process: Applications of transition probability matrix

A Stochastic Process (SP) {*Y* = *Y*_*t*_, *t*∈*T*}, also known as a random process, comprises a series of random variables that are time-indexed. Each element of this set, denoted as {*Y*_*t*_}, is a random variable for a specific time *t* within the index set *(T)*. Depending on whether the index set is continuous or discrete (countable), we refer to the stochastic process as continuous or discrete, respectively. The state space {*Y*_*t*_} can assume it’s all the possible values [30]. The Transition Probability Matrix (TPM) refers to the likelihood of moving or transitioning from one state to another within an SP. These Transition Probabilities (TPs) are essential in modeling and predicting both stationary and non-stationary SPs. The TPM is derived from a Markov Chain (MC), which is an SP {*Y*_*t*_}, retaining the property–”*that the future states of the process depend on the previous states only through the present state of the process* {*Y*_*t*_}*”* [31, 32]. The current approach involves utilizing the Multi-Scalar Seasonally Amalgamated Regional Standardized Precipitation Evapotranspiration Index (MSARSPEI) [19] to determine drought classes on a regional basis, incorporating various timescales and types of drought classification. Specifically, meteorological drought is categorized using timescales 1 and 3, while agricultural drought employs timescales *1*, *3*, and *6*. Hydrological drought, on the other hand, uses timescales *6*, *9*, *12*, *24*, and *48*. These calculations were performed according to [22]. A discrete MC model was utilized to formulate the drought sequences; for instance, the meteorological drought categorized by “*a*” can be considered a drought sequence of timescales *1* and *3*. Similarly, the agricultural and hydrological drought can be considered a drought sequence of timescales *1–6* and *6–48* respectively and formulated in the discrete MC models for both “b” and “c”.

Each particular MC is characterized and marked by a TPM and shows the TPs of drought states (from one state to another). This study assumes a first-order MC, where the drought classes of a future month are conditionally independent, given the drought classes of the current month [24]. Since the TPs are homogeneous over time, we can represent each drought class as a first-order MC. Alternatively, exploring higher-order MC models for drought class time series may be appropriate depending on the previous two or higher-order drought classes. Furthermore, each TPM must meet the following conditions (Eqs 1 and 2) in its mathematical structure;

$$\sum q_{hi}=1$$ (1)

and

$$q_{hi}\leq 1$$ (2)

for all *h* and *i*. The probabilities of transitioning between states are expressed in the form of a matrix, taking into account the following methods:

We can define $X_{hi}^{\left(t\right)}$ as the aggregate count of transitions from one drought state to any other state, within a given number of time steps, *t*. Furthermore, let *X*_*h*_ represents the total number of certain drought states. Therefore, we can use Eq 3 to calculate the probabilities of switching between different drought classes;

$$q_{hi}^{t}=\frac{X_{hi}^{t}}{X_{h}},\;h,i=1,2,3,\ldots ,r$$ (3)

In addition, the following TPM represents the drought classes’ transient behaviour;

$$Q^{\left(t\right)}=[\begin{array}{l} q_{11}^{t}\;q_{12}^{t}\;\ldots \;q_{1r}^{t}\\ q_{21}^{t}\;q_{22}^{t}\;\ldots \;q_{2r}^{t}\\ \vdots \;\vdots \;\vdots \;\vdots \\ q_{r1}^{t}\;q_{r2}^{t}\;\ldots \;q_{rr}^{t} \end{array}]$$

This study considers the states of the SP to be discrete and includes the following drought classes: Moderate Wet (M-W), Very Wet (V-W), Extreme Wet (E-W), Normal Drought (N-D), Moderate Drought (M-D), Severe Drought (S-D), and Extreme Drought (E-D).

## Outlines of the proposed scheme

**Fig 1:**
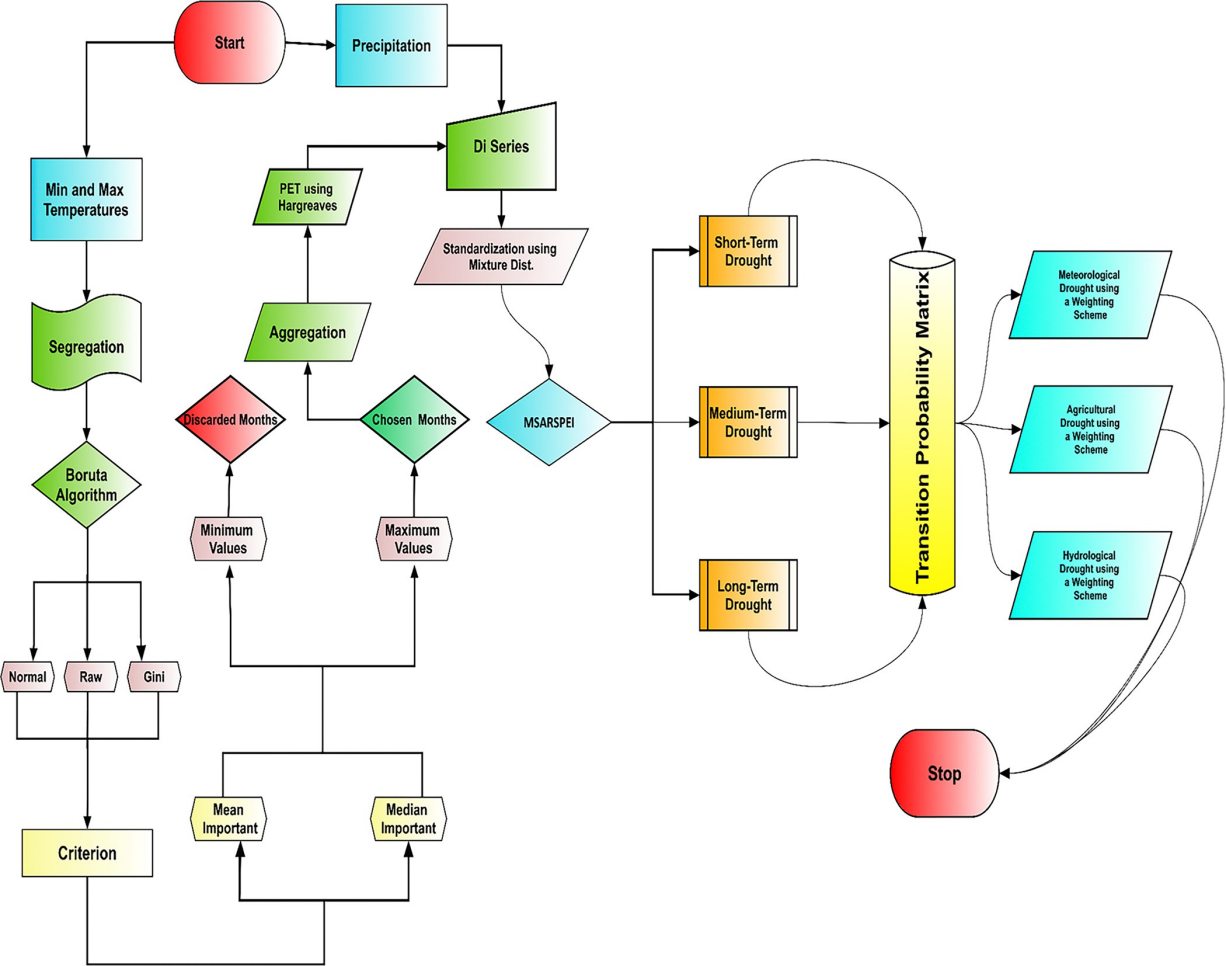
Flowchart of the proposal.

In the current study, we developed a new indicator to utilize various timescales, i.e., *1*, *3*, *6*, *9*, *12*, *24*, and *48* of MSARSPEI (previously discussed in [19], to monitor meteorological (timescales 1–3), agricultural (timescales 1–6) and hydrological (timescale *6–48*) droughts. As the timescales have the same classification states, it is reasonable to use them to evaluate the joint characterization. The proposed mechanism includes the following steps (see Fig 1).

- *Calculation of MSARSPEI*: The first step involves computing MSARSPEI, and for a detailed, step-by-step guide to this calculation, refer to [19].
- *Selection of region and its meteorological stations*: The second step is the selection of the region among *7* regions. In the current study, *Region 4* has been selected randomly. It comprises *9* different meteorological stations.
- *Definition of drought types*: The third step is to define different drought types. According to [33], timescale (1–3), (1–6) and (6–48) represent meteorological, agricultural, and hydrological droughts, respectively. The core objective of this study is to utilize TPM to monitor the said drought types regionally.
- *Introduction of a novel method for decision aggregation*: The fourth step involves introducing a novel method for decision aggregation, which assigns transient probabilities as weights to each drought class at different timescales for each month. For meteorological droughts between timescales 1 and 3, the drought category with the highest weight, as determined by the Switching Probabilities (SwPs), is identified as the joint-determined drought class. A similar weighting scheme is utilized for agricultural and hydrological droughts between timescales 1–6 and 6–48, respectively.

The development of the new scheme is described in the following section.

### Proposed scheme for meteorological, agricultural and hydrological drought

Let TS_1,1_, TS_1,2_, TS_1,3_, …, TS_1,n_, TS_3,1_, TS_3,2_, TS_3,3_,…, TS_3,n_, TS_6,1_, TS_6,2_, TS_6,3_, …, TS_6,n_, TS_9,1_, TS_9,2_, TS_9,3_,…, TS_9,n_, TS_12,1_, TS_12,2_, TS_12,3_, …, TS_12,n_, TS_24,1_, TS_24,2_, TS_24,3_,…, TS_24,n_, and TS_48,1_, TS_48,2_, TS_48,3_,…, TS_48,n_, is the monthly time series record of drought classes estimated from MSARSPEI using timescales *(1–48)* respectively. Furthermore, consider each of the drought-categorized series to be a discrete SP which follows first-order MC. Previous studies have utilized MC models to analyse drought patterns in various climatic regions. For instance, [34–37] and [24] are among the authors who have employed MC models in their research on drought behaviour. In this approach, the temporal classification of timescales is used to determine the transient or dynamic behaviour of a drought category. Consequently, to capture the transient behavior of each drought class across different timescales for meteorological, agricultural, and hydrological droughts, the joint aggregative drought class is identified as the one with the highest SwP. In this instance, the SwPs act as weight factors to assess the suitability of the respective drought class. Thus, under the MC scheme, distinct TPMs are necessary for our proposed framework to determine the likelihood of transitioning from one drought class to another. Matrix 1 presented below illustrates the mathematical structure for selecting SwPs between different drought classes for each drought type.

**Table:** Matrix 1.

Drought Categories_g_	E-W	V-W	M-W	N-N	M-D	S-D	E-D
E-W	p_g11_	p_g12_	p_g13_	p_g14_	p_g15_	p_g16_	p_g17_
V-W	p_g21_	p_g22_	p_g23_	p_g24_	p_g25_	p_g26_	p_g27_
M-W	p_g31_	p_g32_	p_g33_	p_g34_	p_g35_	p_g36_	p_g37_
N-N	p_g41_	p_g42_	p_g43_	p_g44_	p_g45_	p_g46_	p_g47_
M-D	p_g51_	p_g52_	p_g53_	p_g54_	p_g55_	p_g56_	p_g57_
S-D	p_g61_	p_g62_	p_g63_	p_g64_	p_g65_	p_g66_	p_g67_
E-D	p_g71_	p_g72_	p_g73_	p_g74_	p_g75_	p_g76_	p_g77_

Drought characterization is done using the categories of drought given in Table 1 for SDIs.

**Table 1:** Drought categories.

S. No.	SDI Values	Categories
1.	2.00 and above	Extremely Wet
2.	1.50 to 1.99	Very Wet
3.	1.00 to 1.49	Moderate Wet
4.	-0.99 to 0.99	Near Normal
5.	-1.00 to -1.49	Moderate Drought
6.	-1.50 to -1.99	Severe Drought
7.	-2.00 and less	Extremely Drought

**Fig 2:**
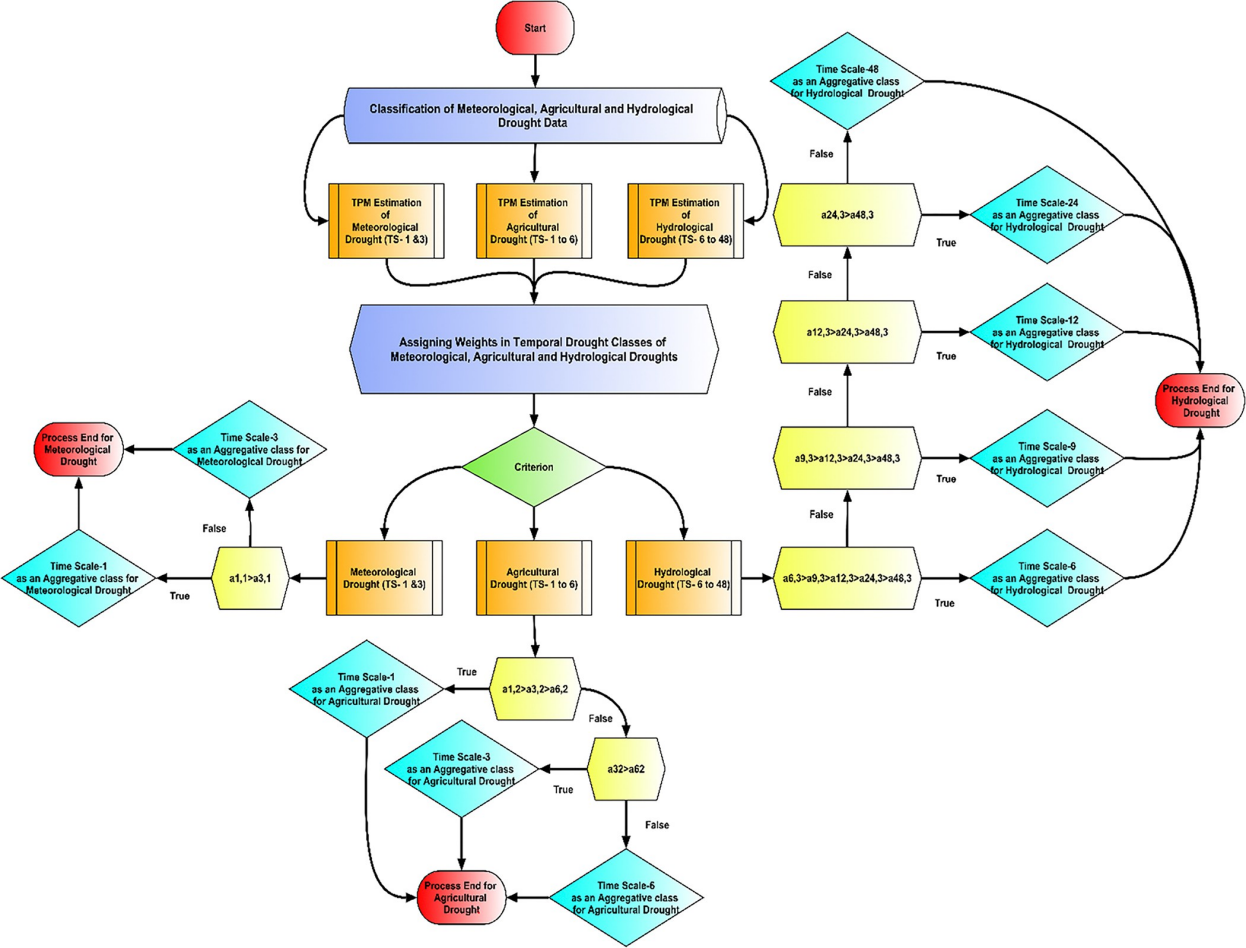
Flowchart of TPM as weights.

In the Markov setting, the SwPs for one-step transitions are denoted by *p*_*gij*_ and are associated with their corresponding transient classes, and *g = 1*, *3*, *6*, *9*, *12*, *24* and *48*, *i* and *j* represent the drought states and drought classes respectively. In the next step, the SwP weights that belong to specific timescale classes, are sorted sequentially. These weights are instrumental in ascertaining the drought category for the following month while accounting for the influence of the previous transient patterns of drought categories in each of the timescales. Table 2 represents the criteria of the weighting schemes for the drought types corresponding to their timescales. Figs 1 and 2 represent a step-by-step procedure of the proposed method.

**Table 2:** Algorithm assessment of temporal drought class using TPM.

Time Scales	Drought Types		
	Meteorological (*a*_*t*,1_)	Agricultural (*a*_*t*,2_)	Hydrological (*a*_*t*,3_)
1	$n_{1,c/c-1},a_{1,1}$	$o_{1,c/c-1},a_{1,2}$	*N*.*A*
3	$n_{1,c/c-1},a_{3,1}$	$o_{1,c/c-1},a_{3,2}$	*N*.*A*
6	*N*.*A*	$o_{1,c/c-1},a_{6,2}$	$p_{1,c/c-1},a_{6,3}$
9	*N*.*A*	*N*.*A*	$p_{1,c/c-1},a_{9,3}$
12	*N*.*A*	*N*.*A*	$p_{1,c/c-1},a_{12,3}$
24	*N*.*A*	*N*.*A*	$p_{1,c/c-1},a_{24,3}$
48	*N*.*A*	*N*.*A*	$p_{1,c/c-1},a_{48,3}$
Proposed Indices	$\max[(n_{1,c/c-1},a_{1,1}),(n_{1,c/c-1},a_{3,1})]$	$\max[(o_{1,c/c-1},a_{1,2}),(o_{1,c/c-1},a_{3,2}),(o_{1,c/c-1},a_{6,2})]$	$\max[(p_{1,c/c-1},a_{6,3}),(p_{1,c/c-1},a_{9,3}),(p_{1,c/c-1},a_{12,3}),(p_{1,c/c-1},a_{24,3}),(p_{1,c/c-1},a_{48,3})]$

## Results and discussion

### Analysis of time series data

To assess the viability of the proposed framework, an analysis was conducted on the time series data of MSARSPEI in Region 4. This region comprises of nine distinct meteorological stations, each operating at different timescales. Table 3 provides a comprehensive overview of the statistical information associated with each of the selected stations. The primary objective of this exercise was to establish the efficacy of the proposed approach while adhering to scientific best practices.

**Table 3:** Statistics of the region under study.

Precipitation (mm) Temperature (*C*°) Coordinates										
Region	Stations	Mean	Min	Max	Mean	Min	Max	Lat (N)	Lon (E)	Altitudes (m)
Bahawalnagar		19.97	4.76	43.60	25.10	23.83	26.58	30.0025°	73.2412°	163
Bahawalpur		15.16	0.95	55.93	25.74	24.63	26.82	29.3544°	71.6911°	214
DI Khan		26.15	11.63	63.03	23.67	22.36	24.77	31.8626°	70.9019°	165
Faisalabad		32.52	14.37	61.23	23.50	22.31	24.72	31.4504°	73.1350°	184
4 Lahore		55.35	21.81	103.92	24.05	22.73	25.04	31.5204°	74.3587°	217
Mianwali		45.56	11.36	90.53	23.70	22.79	24.67	32.6645°	71.4774°	210
Multan		11.97	6.92	42.77	24.88	23.79	26.98	30.1575°	71.5249°	122
Sargodha		39.94	21.13	63.94	23.84	22.83	25.05	32.0740°	72.6861°	190
Sialkot		83.09	45.38	151.23	22.40	21.15	23.48	32.4945°	74.5229°	256

### Methodology for estimating MSARSPEI using long-term time series data

The methodology proposed in this study utilizes long-term time series data of monthly rainfall, as well as maximum and minimum temperatures. For this purpose, the data of said variables are collected from Karachi Data Processing Centre (KDPC) by Pakistan Meteorological Department (PMD). The dataset spans from January 1967 to December 2017, complies with World Meteorological Organization (WMO) requirements, and is error-checked, reviewed, tabulated, and quality controlled by the KDPC. In the estimation process, K-component Gaussian mixture distributions are fitted to calculate MSARSPEI [19].

### Weight determination for each drought class of every timescale

**Fig 3:**
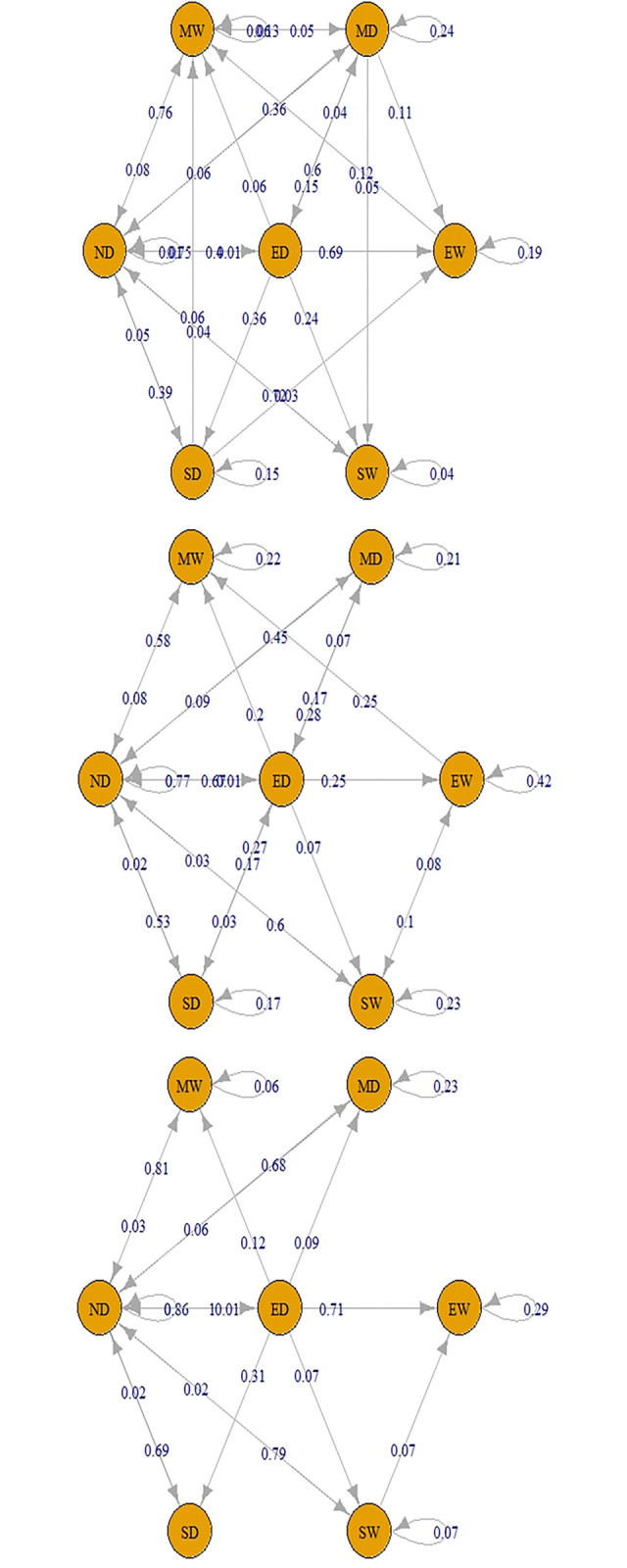
TPM using TS 1–3 for defining Meteorological Drought (a. Short-term drought (TS-1), b. Short-term drought (TS-3), c. Meteorological drought using TPM).

**Fig 4:**
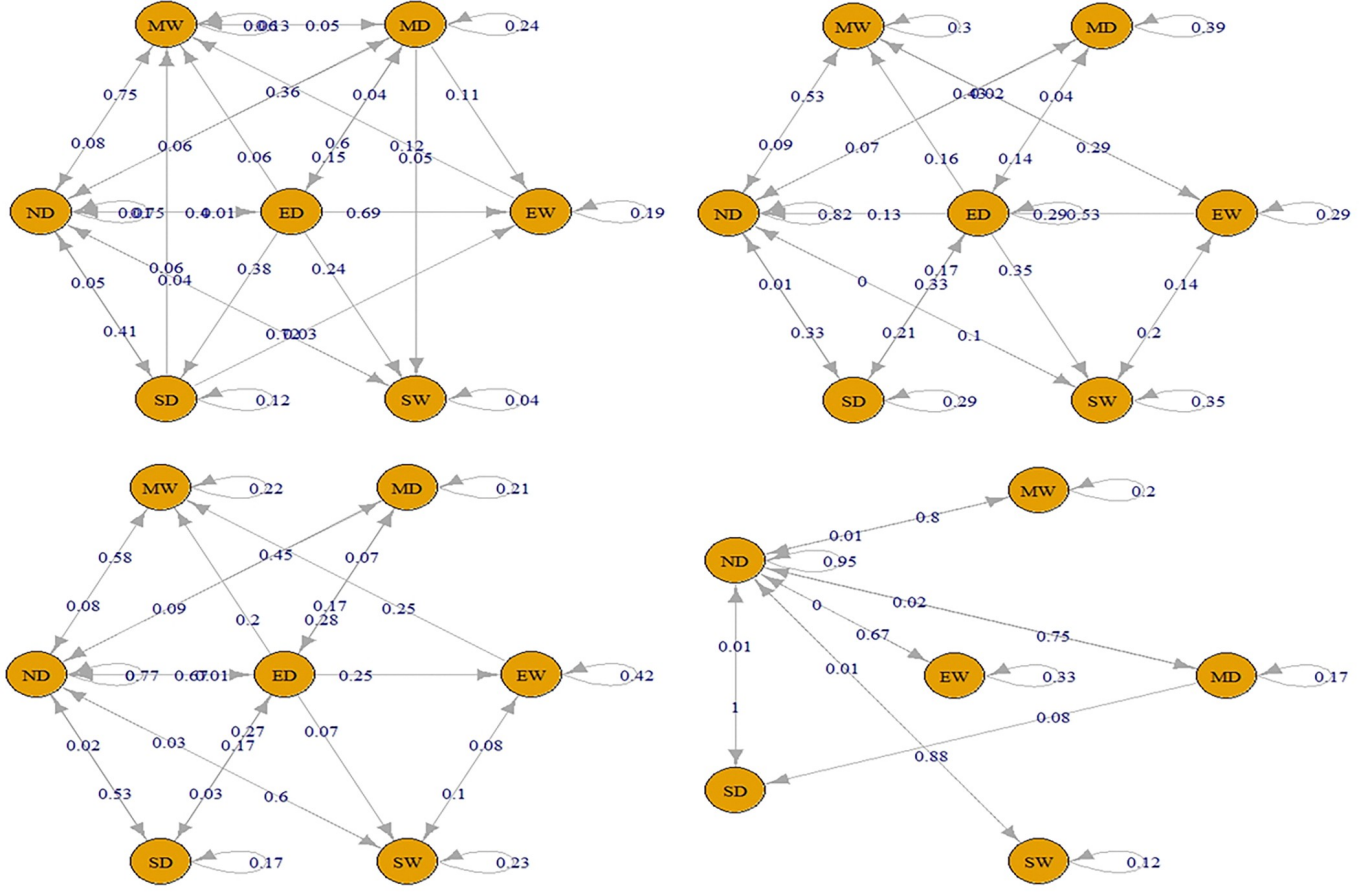
TPM using TS 1–6 for defining Agricultural Drought (a. Medium-term drought (TS-1), b. Medium-term drought (TS-3), c. Medium-term drought (TS-6), d. Agricultural drought using TPM).

**Fig 5:**
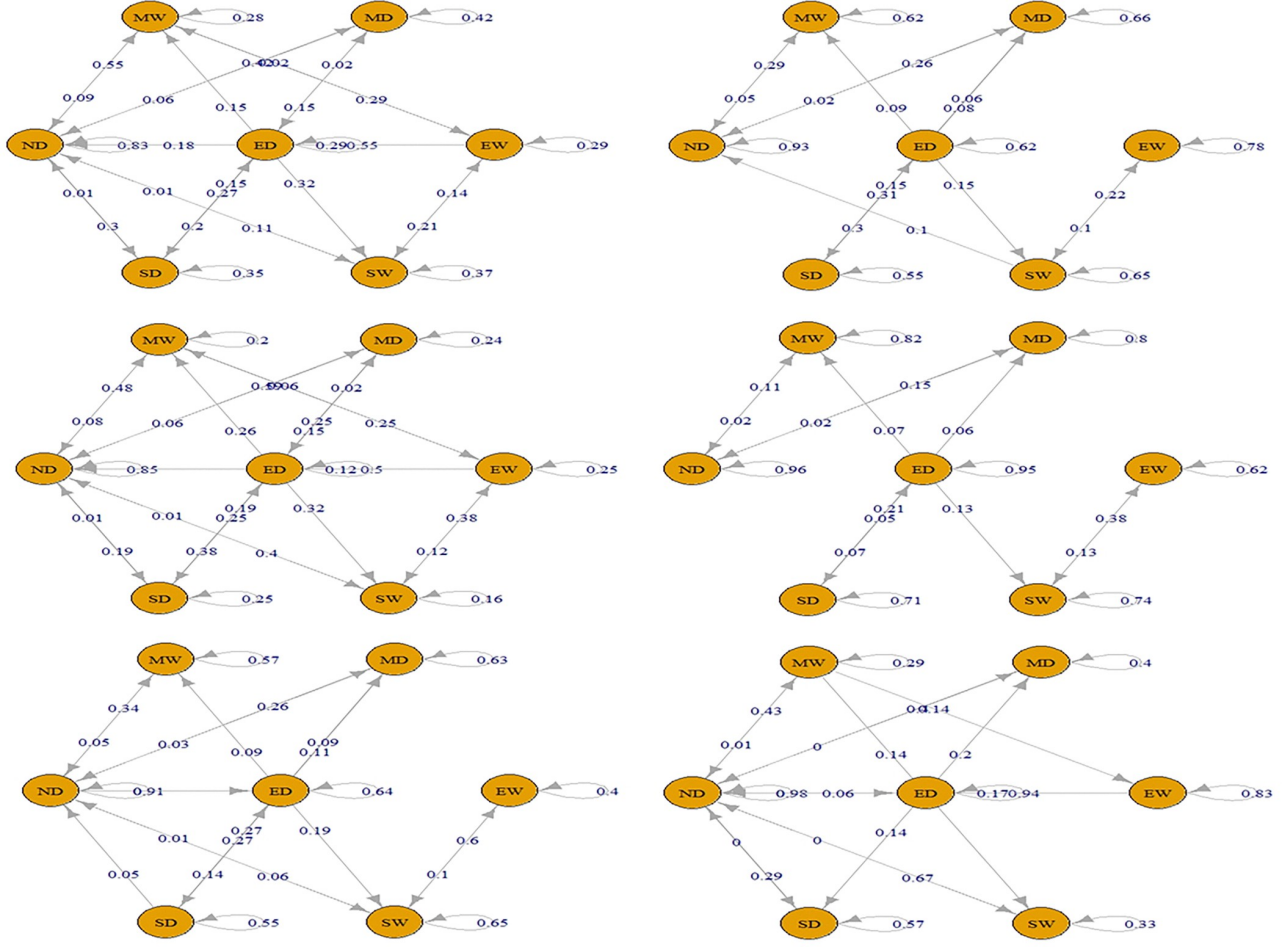
TPM using TS 6–48 for defining Hydrological Drought (a. Long-term drought (TS-6), b. Long-term drought (TS-9), c. Long-term drought (TS-12), d. Long-term drought (TS-24), e. Long-term drought (TS-48), f. Hydrological drought using TPM).

Separate TPMs were created for each drought type to determine the weights. The validity of the first-order MC assumption was tested on small portions of the time series data. This was accomplished by employing the *“verify Markov Property”* function of the Markov chain package in the R language and conducting chi-square tests on these segments. The resulting weights for each drought class of each timescale are depicted in Figs 3–5 illustrating the weights obtained for each drought class of every timescale. A maximum weighting criterion was applied using Eq 3 to address any misclassification of drought classes. TPMs for drought classification series at all timescales are presented in Tables 4 and 5.

**Table 4:** TPM (as weights) of meteorological and agricultural drought.

Drought Types/TS	Drought Categories								
		E-D	E-W	M-D	M-W	N-D	S-D	S-W	sum
Meteorological/1	E-D	**0.000**	0.000	0.600	0.000	0.400	0.000	0.000	1
	E-W	0.000	**0.188**	0.000	0.125	0.688	0.000	0.000	1
	M-D	0.036	0.109	**0.236**	0.055	0.364	0.145	0.055	1
	M-W	0.000	0.000	0.056	**0.130**	0.759	0.000	0.056	1
	N-D	0.007	0.015	0.059	0.081	**0.746**	0.049	0.044	1
	S-D	0.000	0.030	0.364	0.061	0.394	**0.152**	0.000	1
	S-W	0.000	0.000	0.000	0.240	0.720	0.000	**0.040**	1
Meteorological/3	E-D	**0.000**	0.000	0.167	0.000	0.667	0.167	0.000	1
	E-W	0.000	**0.417**	0.000	0.250	0.250	0.000	0.083	1
	M-D	0.069	0.000	**0.207**	0.000	0.448	0.276	0.000	1
	M-W	0.000	0.000	0.000	**0.220**	0.580	0.000	0.200	1
	N-D	0.002	0.010	0.090	0.083	**0.766**	0.019	0.029	1
	S-D	0.033	0.000	0.267	0.000	0.533	**0.167**	0.000	1
	S-W	0.000	0.100	0.000	0.067	0.600	0.000	**0.233**	1
Agricultural/1	E-D	**0.000**	0.000	0.600	0.000	0.400	0.000	0.000	1
	E-W	0.000	**0.188**	0.000	0.125	0.688	0.000	0.000	1
	M-D	0.036	0.109	**0.236**	0.055	0.364	0.145	0.055	1
	M-W	0.000	0.000	0.057	**0.132**	0.755	0.000	0.057	1
	N-D	0.007	0.015	0.059	0.081	**0.748**	0.047	0.044	1
	S-D	0.000	0.031	0.375	0.063	0.406	**0.125**	0.000	1
	S-W	0.000	0.000	0.000	0.240	0.720	0.000	**0.040**	1
Agricultural/3	E-D	**0.000**	0.000	0.167	0.000	0.667	0.167	0.000	1
	E-W	0.000	**0.417**	0.000	0.250	0.250	0.000	0.083	1
	M-D	0.069	0.000	**0.207**	0.000	0.448	0.276	0.000	1
	M-W	0.000	0.000	0.000	**0.220**	0.580	0.000	0.200	1
	N-D	0.002	0.010	0.091	0.083	**0.767**	0.017	0.029	1
	S-D	0.033	0.000	0.267	0.000	0.533	**0.167**	0.000	1
	S-W	0.000	0.100	0.000	0.067	0.600	0.000	**0.233**	1
Agricultural/6	E-D	**0.533**	0.000	0.000	0.000	0.133	0.333	0.000	1
	E-W	0.000	**0.286**	0.000	0.286	0.286	0.000	0.143	1
	M-D	0.036	0.000	**0.393**	0.000	0.429	0.143	0.000	1
	M-W	0.000	0.016	0.000	**0.297**	0.531	0.000	0.156	1
	N-D	0.000	0.000	0.074	0.088	**0.824**	0.010	0.005	1
	S-D	0.208	0.000	0.167	0.000	0.333	**0.292**	0.000	1
	S-W	0.000	0.200	0.000	0.350	0.100	0.000	**0.350**	1

**Table 5:** TPM (as weights) of hydrological drought.

Drought Types/TS	Drought Categories								
		E-D	E-W	M-D	M-W	N-D	S-D	S-W	sum
Hydrological/6	E-D	**0.545**	0.000	0.000	0.000	0.182	0.273	0.000	1
	E-W	0.000	**0.286**	0.000	0.286	0.286	0.000	0.143	1
	M-D	0.021	0.000	**0.417**	0.000	0.417	0.146	0.000	1
	M-W	0.000	0.017	0.000	**0.283**	0.550	0.000	0.150	1
	N-D	0.000	0.000	0.065	0.090	**0.832**	0.008	0.005	1
	S-D	0.200	0.000	0.150	0.000	0.300	**0.350**	0.000	1
	S-W	0.000	0.211	0.000	0.316	0.105	0.000	**0.368**	1
Hydrological/9	E-D	**0.500**	0.000	0.250	0.000	0.000	0.250	0.000	1
	E-W	0.000	**0.250**	0.000	0.250	0.125	0.000	0.375	1
	M-D	0.024	0.000	**0.244**	0.000	0.585	0.146	0.000	1
	M-W	0.000	0.060	0.000	**0.200**	0.480	0.000	0.260	1
	N-D	0.000	0.000	0.061	0.076	**0.846**	0.005	0.013	1
	S-D	0.375	0.000	0.188	0.000	0.188	**0.250**	0.000	1
	S-W	0.000	0.120	0.000	0.320	0.400	0.000	**0.160**	1
Hydrological/12	E-D	**0.636**	0.000	0.091	0.000	0.000	0.273	0.000	1
	E-W	0.000	**0.400**	0.000	0.000	0.000	0.000	0.600	1
	M-D	0.000	0.000	**0.630**	0.000	0.259	0.111	0.000	1
	M-W	0.000	0.000	0.000	**0.566**	0.340	0.000	0.094	1
	N-D	0.003	0.000	0.035	0.045	**0.910**	0.000	0.008	1
	S-D	0.136	0.000	0.273	0.000	0.045	**0.545**	0.000	1
	S-W	0.000	0.097	0.000	0.194	0.065	0.000	**0.645**	1
Hydrological/24	E-D	**0.625**	0.000	0.063	0.000	0.000	0.313	0.000	1
	E-W	0.000	**0.778**	0.000	0.000	0.000	0.000	0.222	1
	M-D	0.000	0.000	**0.658**	0.000	0.263	0.079	0.000	1
	M-W	0.000	0.000	0.000	**0.625**	0.286	0.000	0.089	1
	N-D	0.000	0.000	0.023	0.048	**0.929**	0.000	0.000	1
	S-D	0.300	0.000	0.150	0.000	0.000	**0.550**	0.000	1
	S-W	0.000	0.100	0.000	0.150	0.100	0.000	**0.650**	1
Hydrological/48	E-D	**0.947**	0.000	0.000	0.000	0.000	0.053	0.000	1
	E-W	0.000	**0.625**	0.000	0.000	0.000	0.000	0.375	1
	M-D	0.000	0.000	**0.796**	0.000	0.148	0.056	0.000	1
	M-W	0.000	0.000	0.000	**0.821**	0.107	0.000	0.071	1
	N-D	0.000	0.000	0.019	0.019	**0.963**	0.000	0.000	1
	S-D	0.071	0.000	0.214	0.000	0.000	**0.714**	0.000	1
	S-W	0.000	0.130	0.000	0.130	0.000	0.000	**0.739**	1

**Fig 6:**
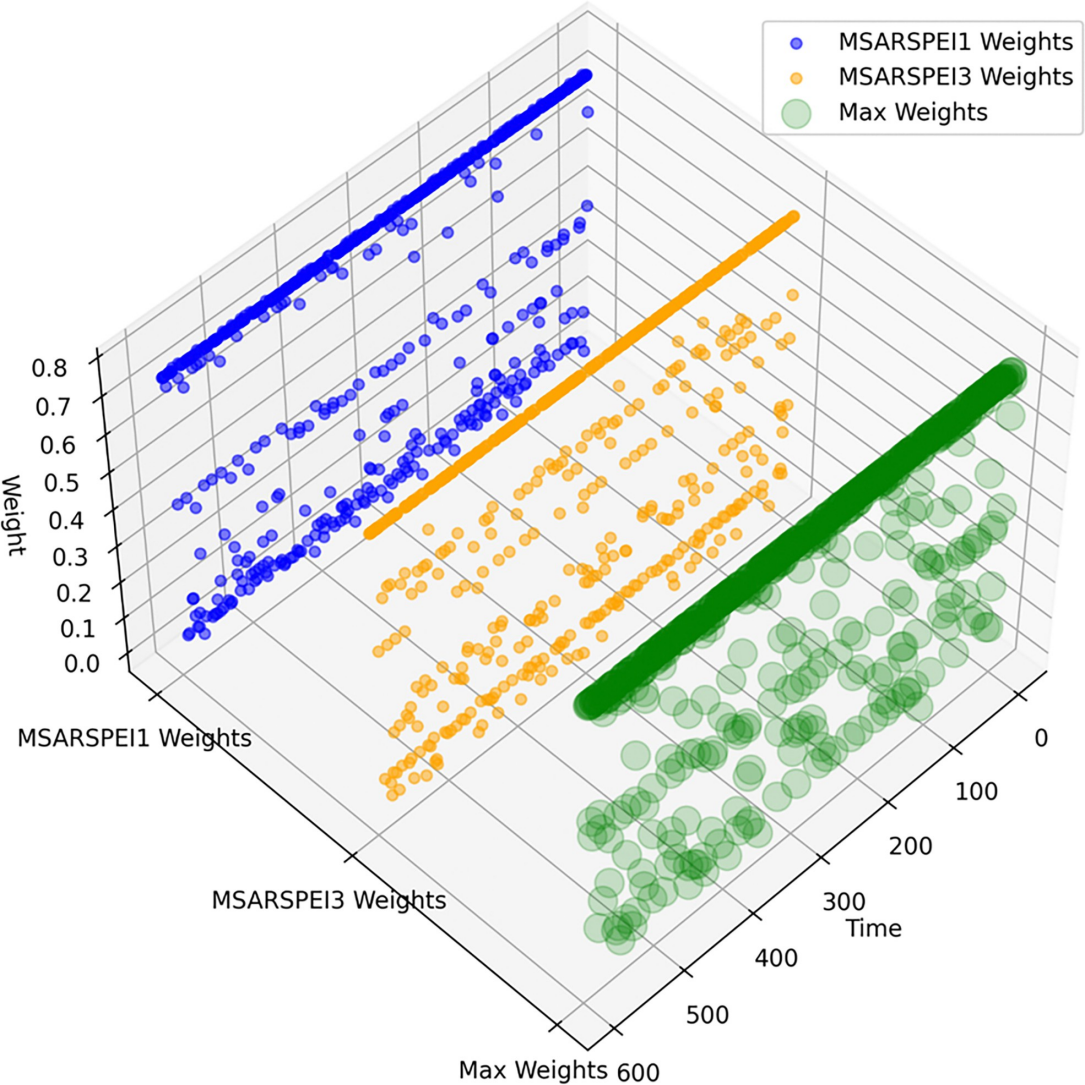
TPM weights.

Furthermore, the graphical representation of the corresponding weights for meteorological drought, MSARSPEI1 and MSARSPEI3 can be observed in Fig 6. The scatter plot in Fig 6 depicts the variation of weights for MSARSPEI1, MSARSPEI3, and the maximum value over time. For instance, the figure shows three scatter plots, each representing one type of weight. If the weight value of MSARSPEI1 is high, it means that this variable has a significant impact on the overall result. Similarly, if the weight value of MSARSPEI3 is low, it means that this variable has a minor impact on the overall result. The meteorological drought is characterized by selecting the maximum weights (TPM) from MSARSPEI1 and MSARSPEI3. Additionally, the same criterion has been taken into account for hydrological and agricultural drought conditions, using their corresponding time scales.

### Investigation of the relationship between drought types and MSARSPEI timescales

**Fig 7:**
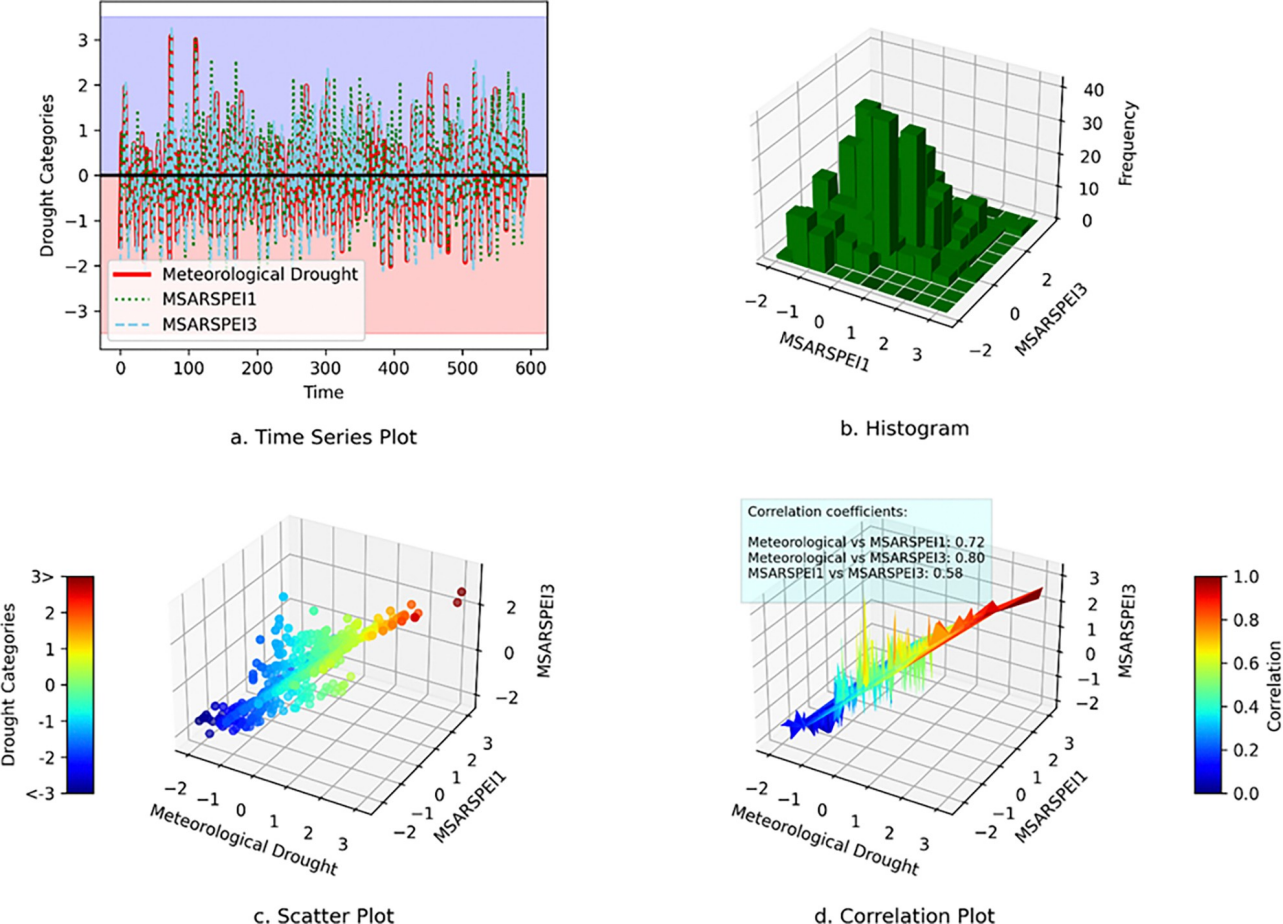
Comparison of meteorological drought with MSARSPEI1 and MSARSPEI3.

In this study, the corresponding TPs for each temporal series of drought type were switched with their corresponding drought class. Additionally, this study investigated the relationship between meteorological drought, agricultural drought and hydrological drought with their corresponding MSARSPEI timescales using various techniques. For instance, the results, as presented in Fig 7a, show three different series representing meteorological drought, MSARSPEI1, and MSARSPEI3, the details can be seen in Fig 7. Further analysis of the data revealed a higher likelihood of mild drought occurrence as shown in Fig 7b and 7c. Finally, Fig 7d indicates a strong positive correlation between meteorological drought and MSARSPEI1/MSARSPEI3, suggesting that the indices can be useful in predicting and monitoring meteorological drought. These findings suggest that the MSARSPEI1 and MSARSPEI3 indices can be valuable tools for meteorological drought monitoring using TPM.

## Concluding remarks

The current study introduces a novel joint aggregative criterion that utilizes multiple timescales to accurately evaluate drought classes, thereby highlighting various types of droughts. In addition, we use temporal switching weights from the TPM to improve drought categorization accuracy. Its efficacy and practical use have been evaluated at nine meteorological stations in Pakistan’s Region 4, demonstrating its practicality in real-world scenarios and assisting in formulating tailored drought mitigation plans. The findings indicate that incorporating several timescales (TS 1–48) can enhance the accuracy and precision of drought monitoring. The analysis led to the following conclusions:

- The use of K-component Gaussian mixture distributions is an effective approach to improve the accuracy of drought categorization. By utilizing this method, the proposed model can better differentiate between different levels of drought severity, which is critical for developing effective drought mitigation policies.
- The implementation of transient memories as weights in the model helps to reduce the error rate in inaccurate drought classes. This approach allows for the model to better adapt to changing weather patterns and account for anomalies in the data, thereby improving the overall accuracy of drought monitoring.
- The strong positive correlation between the proposed meteorological, agricultural, and hydrological droughts with their corresponding timescales provides evidence that the proposed model can effectively capture the dynamics of different types of drought. This supports the validity and reliability of the model in predicting and monitoring drought conditions.
- The use of multiple types of droughts in the proposed model makes it a valuable tool for developing effective drought mitigation policies. By providing a comprehensive view of drought conditions, policymakers can make more informed decisions and allocate resources more effectively to mitigate the impacts of droughts.

In addition, numerical calculations and inferences for other drought indices can also be generalized. In the proposed method, on the other hand, the non-stationary nature of the MC over the entire data length is not considered. Furthermore, the present study considered each MC as a first-order MC in the calculation.

## Supporting information

S1 Data (CSV)
